# Emotion Regulation, Physical Diseases, and Borderline Personality Disorders: Conceptual and Clinical Considerations

**DOI:** 10.3389/fpsyg.2021.567671

**Published:** 2021-02-01

**Authors:** Marco Cavicchioli, Lavinia Barone, Donatella Fiore, Monica Marchini, Paola Pazzano, Pietro Ramella, Ilaria Riccardi, Michele Sanza, Cesare Maffei

**Affiliations:** ^1^Department of Psychology, University “Vita-Salute San Raffaele”, Milan, Italy; ^2^Unit of Clinical Psychology and Psychotherapy, San Raffaele-Turro Hospital, Milan, Italy; ^3^Italian Society for Dialectical Behavior Therapy, Milan, Italy; ^4^Department of Psychology, University of Pavia, Pavia, Italy; ^5^Third Center of Cognitive Psychotherapy – Italian School of Clinical Cognitivism, Rome, Italy; ^6^Villa Azzurra – Psychiatric Specialist Clinic – Neomesia, Riolo Terme, Italy; ^7^Ecopoiesis Centre of Cognitive Psychotherapy of Reggio Calabria, Reggio Calabria, Italy; ^8^Department of Addictions ASL Romagna, Cesena, Italy

**Keywords:** emotion regulation, physical diseases, borderline personality disorder, dialectical behavior therapy, maladaptive behaviors

## Abstract

This perspective paper aims at discussing theoretical principles that could explain how emotion regulation and physical diseases mutually influence each other in the context of borderline personality disorder (BPD). Furthermore, this paper discusses the clinical implications of the functional relationships between emotion regulation, BPD and medical conditions considering dialectical behavior therapy (DBT) as a well-validated therapeutic intervention, which encompasses these issues. The inflexible use of maladaptive emotion regulation strategies (e.g., suppression, experiential avoidance, and rumination) might directly increase the probability of developing physical diseases through a physiological pathway, or indirectly through a behavioral pathway. Some metabolic and chronic medical conditions could significantly impact emotional functioning through biological alterations involved in emotion regulation. Several empirical studies have shown high co-occurrence rates between BPD and several chronic physical diseases, especially ones linked to emotion-based maladaptive behaviors. DBT addresses physical diseases reported by individuals with BPD reducing problematic behaviors functionally associated to emotion dysregulation and identifying physical health as a goal for *Building a Life Worth Living*.

## Introduction

The influence of emotional processes on physical health has long been hypothesized and investigated. For instance, Hippocrates affirmed that the bodily humors, which represent the basis of personality and related emotional experiences, influence diseases (Allport, 1961). In the mid-20th century, psychoanalysts hypothesized that psychological conflicts could be involved in the development of somatic symptoms that represent the manifestation of underlying repressed unacceptable feelings and emotions (e.g., Alexander et al., 1968). Similarly, Selye (1955), who coined the term “stress,” empirically investigated the physiological impacts of emotional reactions to environmental demands and their possible links to physical health. Accordingly, it has been historically assumed that emotional difficulties play a causal role in diseases (Kubzansky and Winning, 2016). However, the onset and persistence of different physical diseases might also significantly affect the quality of emotional reactions and the ability to manage them (De Ridder et al., 2008).

Therefore, the current perspective paper aims at discussing theoretical principles that could explain how emotion regulation and physical diseases mutually influence each other. Accordingly, a functional behavioral approach (for an overview of core principles see: Sugai et al., 2000) is adopted to clarify: (a) how difficulties with emotion regulation and related behaviors increase the probability of developing and maintaining diseases, and (b) how physical diseases constitute relevant vulnerability factors for engaging in maladaptive emotion regulation strategies. Furthermore, the therapeutic implications of this approach are discussed. Particularly, we consider the clinical condition borderline personality disorder (BPD), which is well-recognized to be characterized by emotion dysregulation and high comorbid rates with several chronic medical conditions. Furthermore, the paper discussed the clinical utility of dialectical behavior therapy (DBT; Linehan, 1993), which is a third-wave cognitive-behavioral intervention (Masuda and Rizvi, 2019) based on the application of mindfulness principles (Robins, 2002) specifically developed to treat BPD, as an evidence-based approach (Panos et al., 2014) to effectively address the maladaptive associations between emotional dysregulation and medical diseases.

According to these aims, the current perspective paper was structured as follows. First of all, several models of emotion regulation were discussed in order to provide a conceptual framework to identify adaptive and maladaptive processes. Subsequently, associations between maladaptive emotion regulation and physical diseases were reviewed in order to highlight how these aspects mutually influence each other. Starting from this evidence, the work described the key role of difficulties in emotion regulation as core features of BPD that could explain its clinical manifestations and its high comorbid rates with several chronic medical conditions. Ultimately, the main principles of DBT were discussed in terms of how this structured therapeutic framework could be applied to address difficulties in emotion regulation, their relationship with maladaptive behaviors and the consequences of physical diseases among individuals with BPD.

### Emotion Regulation Processes

Although there is not a definitive consensus on operationalizing emotion regulation (e.g., Cole et al., 2004), different theoretical perspectives refer to this construct by considering all the processes involved in consciously or automatically influencing positive and negative emotions, in terms of their intensity, duration, and/or quality (Naragon-Gainey et al., 2017). With respect to this broad conceptualization, Naragon-Gainey et al. (2017) identified three groups of theoretical models of emotion regulation. The temporal process model of emotion regulation (e.g., Gross, 1998, 2015) posits that it can be implemented at any of the four stages of emotion generation—an emotion is elicited by a situation, attention to contextual features of situation, cognitive appraisal of the situation and emotional response (i.e., behavioral, physiological, and experiential). Accordingly, the temporal process model of emotion regulation yields specific emotion regulation strategies for each stage of emotion generation, namely situation selection (e.g., avoidance of environmental contexts altogether) and situation modification (e.g., changing or avoiding specific features of a situation), attentional deployment (e.g., distraction), cognitive change (e.g., reappraisal), and response modulation (e.g., expressive suppression). Strategy-based models of emotion regulation (e.g., Aldao et al., 2010; Aldao and Nolen-Hoeksema, 2012) recognize “adaptive” (e.g., acceptance, problem solving, mindfulness) and “maladaptive” (e.g., experiential avoidance [EA], rumination, worry) emotion regulation strategies in the light of their positive and negative relationships with psychopathological symptoms, respectively. Focusing on the maladaptive mechanisms of emotion regulation, EA has two related parts: (a) unwillingness to remain in contact with aversive private experiences (e.g., bodily sensations, emotions, thoughts, memories, and behavioral predispositions), and (b) action taken to alter aversive experiences or the events that elicit them (Chawla and Ostafin, 2007). Rumination describes “*a mode of responding to distress that involves repetitively and passively focusing on symptoms of distress and on the possible causes and consequences of these symptoms*” (Nolen-Hoeksema et al., 2008; p. 400). Worry is largely considered a specific form of rumination focused on anxious symptoms (Watkins et al., 2005).

**FIGURE 1:**
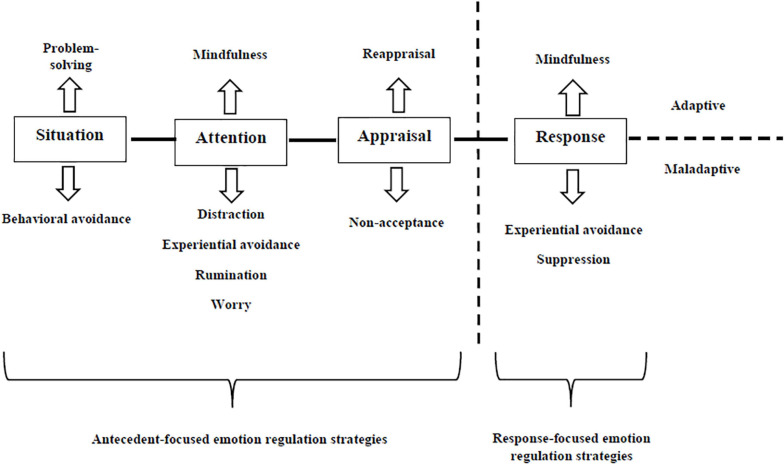
An integration of temporal process and strategy-based models of emotion regulation.

Additionally, Naragon-Gainey et al. (2017) have described a third group of emotion regulation models, ability-based models (e.g., Gratz and Roemer, 2004; Berking et al., 2008) that capture dispositional abilities — emotion recognition, responses to emotions, modulation of emotion-based behaviors, perceived access to effective emotion regulation strategies — involved in different emotion regulation strategies and situations, which are hypothesized to facilitate healthy emotion regulation and to maintain psychopathological conditions. Figure 1 provides an integration of temporal process and strategy-based models of emotion regulation based on the meta-analytic results of Naragon-Gainey et al. (2017).

### Reciprocal Influences Between Emotion Regulation and Physical Diseases

Empirical research has shown significant relationships between several emotion regulation processes and different physical diseases. For instance, there is a growing consensus that supports the impact of negative affectivity in the development of cardiovascular diseases (CVD) and coronary heart diseases (CHD) (e.g., Roest et al., 2010; Kupper and Denollet, 2018). These findings are largely consistent with several studies that have found that suppression, which is linked to persistent negative emotionality (e.g., Wegner and Zanakos, 1994; Moore et al., 2008), was rigidly used by individuals with CVD and CHD (Mund and Mitte, 2012). Furthermore, additional maladaptive emotion regulation strategies, such as worry and rumination were also associated to the previous clinical conditions (Tully et al., 2013). Similar findings were highlighted among individuals with chronic pain. Specifically, the systematic review published by Koechlin et al. (2018) based on the results of 15 studies showed that individuals affected by chronic pain reported a recurrent use of response-focused emotion regulation strategies (e.g., expressive suppression, EA), which were associated to the onset and maintenance of this physical disease.

Therefore, this evidence has highlighted that several maladaptive emotion regulation processes, especially related to dysfunctional attention mechanisms (i.e., rumination and worry) and response-focused strategies (i.e., suppression and EA), played a role in clarifying the onset and maintenance of CVD, CHD, and chronic pain. Accordingly, these findings might suggest that maladaptive emotion regulation processes have a direct impact on physical diseases. This direct link might be partially explained considering the effects of these emotion regulation strategies on the activation of the hypothalamic–pituitary–adrenal (HPA) axis and the sympathetic nervous system, which increase inflammatory mechanisms associated with the subsequent onset of physical conditions (e.g., Appleton and Kubzansky, 2014).

However, dysfunctional emotion regulation strategies could also represent distal risk factors for physical diseases, considering their functional associations with unhealthy behaviors. Individuals frequently engage in maladaptive behaviors, such as binge eating or substance abuse (e.g., alcohol, tobacco, and drugs), in order to avoid, suppress or control different emotional states (e.g., Chawla and Ostafin, 2007; Lavender et al., 2015; Dingemans et al., 2017). Several empirical studies have consistently demonstrated that such behaviors represent relevant risk factors for the onset of CVD, CHD, chronic pain and other medical conditions (e.g., asthma, diabetes, gastrointestinal, and sleep problems/disorders) (Olguin et al., 2017; Thornton et al., 2017; Walker and Druss, 2018).

**FIGURE 2:**
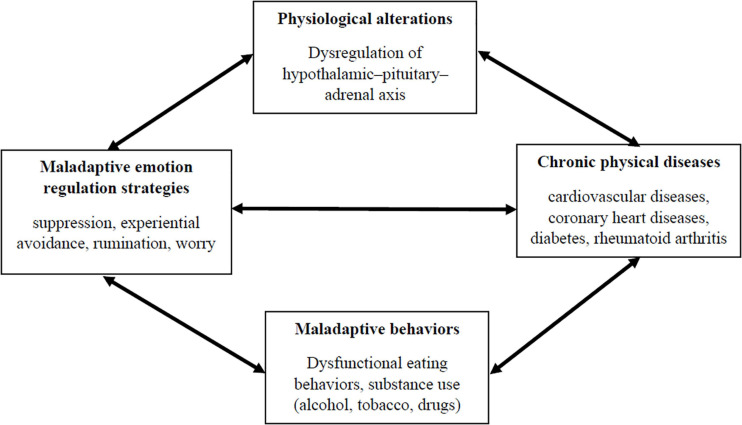
The mutual relationships between emotion regulation and physical diseases.

Hence, the inflexible use of maladaptive emotion regulation strategies — suppression, EA, rumination, worry — might directly increase the probability of developing physical diseases through a physiological pathway (e.g., heightened activity of HPA), or indirectly through a behavioral pathway. However, it has also been demonstrated that people with chronic medical conditions have an elevated risk of reporting emotional difficulties. Indeed, results from meta-analytic studies have indicated that people with different chronic physical conditions (e.g., diabetes, hypertension, rheumatoid arthritis) have a higher risk of developing depressive symptoms compared to healthy individuals (e.g., Dickens et al., 2002; Rotella and Mannucci, 2013; Li et al., 2015). Similar findings were also replicated when the impact of several chronic medical diseases on the onset of anxiety-related conditions were considered (e.g., Smith et al., 2013; Pan et al., 2015). This evidence has led some scholars to hypothesize shared biological mechanisms between physical and affective conditions. For instance, Moulton et al. (2015) suggested that type two diabetes causes an overactivation of innate immunity that leads to a cytokine-mediated inflammatory response and a dysregulation of the HPA axis. These biological reactions might directly affect the brain, causing depressive symptoms. The dysregulation of the HPA axis induced by CHD was also linked to the development of depressive and anxious symptoms frequently reported by individuals with these medical conditions (e.g., Grippo and Johnson, 2002). Consistently with this evidence, it could be possible to conclude that some metabolic and chronic medical conditions could significantly impact the emotional functioning of individuals through biological alterations of the HPA axis, which is considered a valid biomarker of emotion dysregulation (e.g., Braquehais et al., 2012). Figure 2 summarizes pathways that link emotion regulation mechanisms to physical diseases.

### Borderline Personality Disorder: Emotion Dysregulation and Maladaptive Emotion Regulation Strategies

Borderline personality disorder is a severe mental disorder characterized by dysfunctions in a wide range of emotional, behavioral, interpersonal, and cognitive processes (American Psychiatric Association [APA], 2013). The second edition of the Psychodynamic Diagnostic Manual (Lingiardi and McWilliams, 2017) provides a useful clinical description of each domain of functioning in BPD. With respect to the emotional domain, individuals with BPD are described as persons that feel “*emotions that easily spiral out of control and reach extremes of intensity, compromising their capacity for adaptive functioning* (Lingidardi and McWilliams p. 53)”. Intense affective reactions of being rejected and abandoned characterized the interpersonal functioning of individuals with BPD, especially regarding close relationships. These intense emotional states dispose individuals with BPD to misunderstand the attitudes and behaviors of others as signs of present or future rejection and abandonment. Their interpersonal functioning is also characterized by broad impairments in mentalization, which are reflected in frequent misinterpretation of other people’s behaviors, intentions, and emotions together with difficulties in taking other perspectives. The rapid oscillations of emotions and self-other representation affect the sense of continuity of their own experience. The emotional turmoil is strictly connected with the behavioral domain of BPD. Individuals with BPD use self-mutilating behavior to soothe themselves, feel alive or reconnect with their bodies. Individuals with BPD may make suicide threats or gestures for the same reasons. Suicide attempts might also have an interpersonal function as a form of manipulation of others together with the intention to attract other people’s attention. Ultimately, problematic sexual and aggressive behaviors that characterized individuals with BPD could be linked to interpersonal relationships, especially when attachment needs are stirred up. The dysfunctional relationships existing among the emotional, cognitive, behavioral and interpersonal domains might explain how individuals with BPD tend to have trouble making and maintaining long-lasting, gratifying close relationships and stable, satisfying work lives.

Starting from this clinical description, several clinicians have proposed different etiopathogenetic theories of BPD (for a review see: Gunderson et al., 2018). One of the most influential clinical theories of BPD was proposed by Linehan (1993), who hypothesized that the core feature of the disorder is emotional dysregulation. Consistently with this model, BPD emotion dysregulation was considered the result of repetitive transactions between an individual biological vulnerability and the effects of invalidating environments (Crowell et al., 2009). The biological vulnerability includes three different, albeit interrelated, characteristics of emotional response: (a) hyper-sensitivity — low threshold for emotional reactions; (b) hyper-reactivity — intense emotional responses; (c) slow return to emotional baseline — long-lasting emotional reactions. Several experimental studies have provided support for this conceptualization of emotion dysregulation among individuals with BPD (for meta-analytic reviews see: Ruocco et al., 2013; Mitchell et al., 2014; Kaiser et al., 2016; Schulze et al., 2016, 2019; Bortolla et al., 2020). However, other scholars (e.g., Selby and Joiner, 2009; Chapman et al., 2011; Carpenter and Trull, 2013) have developed Linehan’s original formulation of emotion dysregulation. These authors have sustained that several maladaptive emotion regulation strategies play a key role in sustaining the biological alterations of Linehan’s biosocial model and in explaining core dysfunctional behaviors in BPD, such as suicidal and non-suicidal self-injury behaviors, substance abuse and other problematic eating behaviors. According to these hypotheses, empirical research has shown that individuals with BPD rigidly use response-focused emotion regulation strategies (i.e., suppression, EA and dissociation) (for meta-analytic reviews see: Cavicchioli et al., 2015; Scalabrini et al., 2017) and other emotion regulation strategies linked to altered attentional mechanisms, such as rumination and worry (Cavicchioli and Maffei, 2020, under review). Several studies have also demonstrated that all these strategies have deleterious effects on emotional responses, chiefly increasing their intensity and duration (e.g., Sloan, 2004; Barnow et al., 2012; LeMoult et al., 2013). Furthermore, empirical evidence has shown that these maladaptive emotion regulation strategies represent significant mediators of relationships between BPD emotional vulnerabilities and core dysfunctional behaviors, especially self-harm, binge eating and substance-use behaviors (e.g., Chapman et al., 2005; Selby et al., 2009).

Taking these findings together, emotion dysregulation in BPD should be conceptualized taking into account two interrelated levels. The first level refers to biological alterations of emotional response. The second level is related to the inflexible use of maladaptive response-focused emotion regulation strategies together with the engagement in dysfunctional attentional emotion regulation processes. These levels are mutually influenced and, they are reinforced through core problematic behaviors in BPD. The interdependence among such mechanisms over time might explain the chronic and pervasive dysregulation of the system of emotions characterizing individuals with BPD.

### Borderline Personality Disorder and Physical Diseases: Empirical Evidence

Taking together the direct and indirect effects of maladaptive emotion regulation strategies on the onset and maintenance of several chronic physical diseases and the key role of emotion dysregulation in BPD, it could be hypothesized that individuals with BPD have an elevated risk of developing comorbid medical conditions. Considering the direct physiological pathway linking maladaptive emotion regulation to physical diseases through alterations of the HPA axis, several studies have demonstrated persistent abnormalities in the HPA axis among individuals with BPD, especially for those with other comorbid conditions such as depressive and posttraumatic stress disorders (PTSD) (e.g., Zimmerman and Choi-Kain, 2009). With respect to the indirect pathway sustained by frequent engagement in unhealthy behaviors (e.g., substance use and maladaptive eating behaviors), high co-occurrence rates between BPD and substance use disorders are well-documented (22% across different settings), especially alcohol (17%), cocaine (22%), and opioid (34%) use disorders (Trull et al., 2018). The significant associations between BPD and eating disorders have also been supported. A recent meta-analysis showed that up to 20% of individuals with anorexia and bulimia nervosa meet criteria for BPD (Martinussen et al., 2017). Similar results were found considering the portion of individuals with binge eating disorder and BPD (e.g., Sansone et al., 2004, 2008).

Empirical evidence seems to confirm the hypothesis linking BPD to an elevated risk of developing several physical diseases. Considering CVD, individuals with BPD showed a risk of reporting ischemic heart disease and stroke that was eight times higher than non-BPD subjects (Moran et al., 2007). The elevated risk of reporting CVD in later adulthood among patients with BPD was replicated in another large sample and, it was explained by a mediating role of obesity, which is significantly associated to maladaptive eating behaviors frequently reported by individuals with BPD (Powers and Oltmanns, 2013). The high comorbidity with metabolic syndromes (e.g., hyperglycemia and hypertriglyceridemia) related to obesity was also confirmed among individuals with BPD, showing a two-fold risk over non-BPD subjects (Kahl et al., 2013). Furthermore, El-Gabalawy et al. (2010) conducted an epidemiological study using a nationally representative sample (*N* = 34,653) evaluating associations between BPD and different physical conditions — arteriosclerosis or hypertension; hepatic disease; diabetes; cardiovascular disease; venereal disease; gastrointestinal disease; obesity; arthritis; and stroke. Results showed that up to 50% of individuals with BPD suffered from at least one of these medical conditions. The most recurrent physical diseases were obesity (33.6%), arteriosclerosis or hypertension (28.2%) and arthritis (27.7%), followed by cardiovascular disease (15.3%) and gastrointestinal disease (12.1%). Interestingly, the analyses also showed an elevated risk of developing the medical conditions previously mentioned (i.e., adjusted odd ratios ranged from 1.46 [1.21–1.78] to 2.80 [1.80–4.36]), even controlling for sociodemographic and clinical variables (i.e., any anxiety, mood, or substance use disorders (SUDs), and any personality disorder). Ultimately, Quirk et al. (2016) reviewed results from 13 population-based studies examining the prevalence of personality disorders and their associations with physical comorbidities across different continents (i.e., North America, Western Europe, and Oceania). The results of this review further confirmed a specific association between BPD and CVD. An additional medical condition significantly associated with BPD is chronic pain. With respect to this physical disease, Sansone and Sansone (2012) summarized results of eight studies showing that roughly 30% of patients with chronic pain met the criteria for BPD and, individuals with BPD consistently reported higher levels of pain than those without this disorder. More recently, several empirical studies have replicated the significant association between BPD and chronic pain (e.g., Reynolds et al., 2018; Reynolds and Tragesser, 2019; Johnson et al., 2020).

**FIGURE 3:**
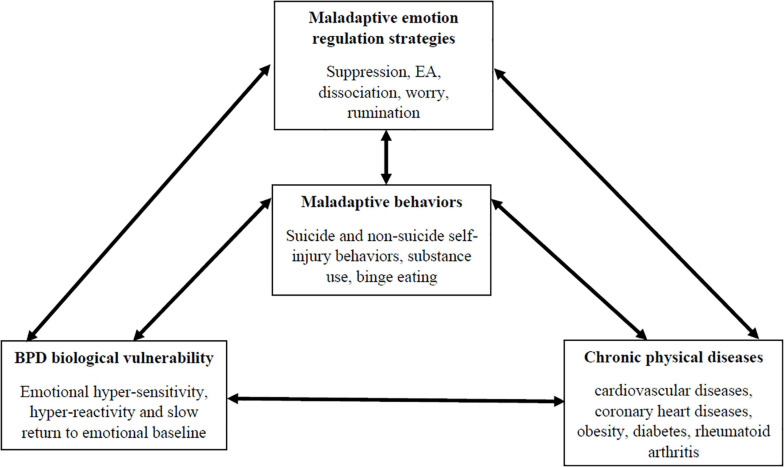
The relationships between emotional functioning of individuals with BPD and physical diseases.

Therefore, this evidence consistently supports that individuals with BPD have an elevated risk of developing severe chronic physical diseases, especially CVD, obesity-related conditions and chronic pain. According to the hypotheses mentioned above, this elevated risk might be explained by a direct effect of emotion dysregulation on alterations of the HPA axis together with the indirect effects of maladaptive behaviors robustly associated to different dysfunctional emotion regulation strategies. However, taking into account the biological effects of these medical conditions on emotional functioning (Grippo and Johnson, 2002), they should be considered relevant factors involved in sustained emotion dysregulation in BPD. Hence, medical conditions should be included in clarifying mechanisms determining the broad difficulties in regulating emotion reporting by BPD patients. Consistently, a comprehensive assessment of medical conditions becomes one of the primary target of BPD treatments, and the adequate resolution of physical diseases should be considered an objective of psychotherapeutic interventions for BPD in the light of their impact on the core features of the disorder. Figure 3 depicts the functional relationships between emotion dysregulation and physical diseases in BPD.

### Dialectical Behavior Therapy for the Treatment of BPD: Core Principles

Dialectical behavior therapy was originally developed as an attempt to apply behavioral principles for the treatment of highly suicidal women (Linehan, 1981). Currently, the latest meta-analytic review (Cristea et al., 2017) aggregating the results of 13 randomized controlled trials evaluating the effects of DBT for BPD highlighted that this therapeutic model should be considered one the most effective treatments for this disorder with the largest empirical support of efficacy. From its origin, DBT has been modified using a trial-and-error approach based on problem solving and social learning theory for addressing clinical problems of highly suicidal and BPD patients (Linehan and Wilks, 2015). However, the exclusive use of cognitive-behavioral strategies has produced invalidating experiences in clients, causing hostility toward therapists or recurrent termination of treatment. Consequently, acceptance and distress tolerance strategies have been combined with change strategies and suggestions for a dialectical use of them. Accordingly, Linehan has introduced mindfulness behavioral skills that could be taught to both clients and therapists (Linehan and Wilks, 2015).

Dialectical behavior therapy treatment includes various modes — individual psychotherapy, group skills training, telephone coaching, and team consultation. Individual therapy primarily aims to reinforce motivation for change and to overcome obstacles to achieving personal goals and values. DBT skills training is delivered in a learning group format and has the purpose of helping patients substitute maladaptive behaviors with goal-oriented behaviors based on four skill modules: mindfulness, emotional regulation, interpersonal effectiveness, and distress tolerance (Linehan, 1993). The main role of telephone coaching is helping clients to use skills in their everyday lives between sessions, supporting the learning and generalization of DBT skills in different contexts and environments. Ultimately, the weekly consultation team ensures adherence to the treatment model, prevents therapist burnout, and provides additional support to those patients who are at high risk of engaging in severely dysfunctional behaviors.

The main treatment goal of DBT is to “*build a life worth living*” (Linehan, 1993; Coyle et al., 2019). Considering the clinical complexity of individuals with BPD, DBT provides guidelines for clinicians to prioritize problems within sessions in order to effectively achieve the main treatment goal. Accordingly, targets of the treatment are grouped into four stages. Stage 1 focuses on clients ceasing problematic behaviors by improving their use of adaptive skills. The behavioral targets are hierarchically organized as follows: (i) decrease imminent life interfering behaviors (e.g., suicide attempts, and non-suicidal self-injury); (ii) reduce therapy interfering behaviors (e.g., missing treatment and behaviors that burn out the therapist); (iii) decrease quality-of-life interfering behaviors (e.g., substance use, unemployment, and homelessness); (iv) increase behavioral skills. The second stage is called “quiet desperation.” Targets of stage 2 are improving the quality of emotional experiences and the treatment of PTSD. Stage 3 is aimed at reducing ordinary problems in living. Finally, stage 4 is provided to increase a sense of completeness and/or achieve transcendence.

Consistently with the hierarchical organization of the treatment, DBT postulates a thorough assessment of clinical problems through functional behavioral analysis (FBA), which is also called “*chain analysis*.” FBA refers to a systematic process of understanding factors that contribute to and maintain a specific behavior (e.g., Horner, 1994). The conceptual assumptions of FBA have their roots in the work of the early behaviorists and in principles of operant and classical conditioning (for a review see: Baum, 2004). Specifically, FBA attempts to identify which internal and external contingencies (e.g., rewards and punishments) maintain a behavior and which change it. Chain analysis is largely used in the early stages of treatment when clients engage in problematic behaviors. Specifically, a clinician conducts a chain analysis following the hierarchy of target behaviors. FBA in DBT provides a complete sequence of events, including both internal and external cues and triggers that lead to target behaviors. Furthermore, the chain analysis is composed of five key elements that a therapist must consider for a comprehensive and effective behavioral assessment: vulnerability factors, prompting events, links on the chain, problem behaviors, and consequences (Rizvi and Ritschel, 2014).

### The Inclusion of Physical Diseases in Treating BPD With DBT

Summarizing the evidence previously discussed, a large portion of individuals with BPD suffer from severe and chronic physical diseases (i.e., CVD, obesity-related conditions, and chronic pain). These medical conditions are robustly associated with problematic behaviors (e.g., substance abuse and binge eating) linked to emotion dysregulation in BPD. Furthermore, severe and chronic physical problems represent relevant biological factors that interfere with adaptive emotional functioning. Consistently, physical health should be considered a key aspect within psychotherapy interventions, especially for BPD.

With respect to DBT, a therapist focuses on physical diseases and related behaviors in different stages of treatment. Independently of the stage in the therapy, chain analysis is the key therapeutic tool to effectively clarify the functional relationships between medical conditions and different clinical features of BPD. During stage 1, the onset and maintenance of a specific physical condition, such as CVD, might be treated as a consequence of the recurrent engagement in several quality-of-life interfering behaviors, especially substance abuse and binge eating. With respect to this scenario, the clinician’s and patient’s primary treatment goal in individual psychotherapy is to work on improving the effective use of behavioral skills learned during the group DBT skills training. Mindfulness skills (e.g., *observing non-judgmentally and being effective*) (Linehan, 2014; pp. 151–213) and behavioral abilities (e.g., *STOP, TIP, pros and cons, distraction, self-soothe with five senses, dialectical abstinence*, and *clear mind*) (Linehan, 2014; pp. 419–490) included in the distress tolerance module might be particularly appropriate for reducing or ceasing these problematic behaviors and consequently, they might address factors that sustain related medical conditions.

Alternatively, it is possible that physical conditions, especially chronic pain (Kirtley et al., 2020), might be the prompting event for other life-interfering behaviors in BPD (i.e., suicide attempts and non-suicidal self-injury behaviors), as empirically demonstrated in epidemiological studies (e.g., Druss and Pincus, 2000; Singhal et al., 2014). Considering this functional link between the onset of medical diseases and the engagement in these problematic behaviors, the primary goal of DBT intervention might be to reinforce emotion regulation skills (e.g., *checking the fact, problem solving, accumulating positive emotions*, and *mindfulness of current emotions*), within both individual and group settings, (Linehan, 2014; pp. 350–408) and reality acceptance skills (e.g., *radical acceptance, turning the mind*, and *willingness*) included in the distress tolerance module (Linehan, 2014; pp. 451–469). The improvement of these cognitive-behavioral skills should help individuals with BPD and chronic pain to effectively regulate distressing, justified emotions elicited and sustained by a severe medical condition without making it worse through life interfering behaviors.

Conducting a chain analysis, the patient and the therapist might also highlight the role of physical diseases as significant vulnerability factors for difficulties in regulating emotions. Several studies have demonstrated that chronic pain and different cardiovascular and metabolic diseases linked to obesity significantly alter the functioning of HPA (e.g., McBeth et al., 2005; Pasquali et al., 2006; Bose et al., 2009; Ortiz et al., 2020). According to the DBT model and this empirical evidence, BPD vulnerability to emotional dysregulation — heightened sensitivity and reactivity to emotional stimuli, together with a slow return to the emotional baseline — might be sustained by an inadequate management of physical illness in the light of intense fear of going to a physician or behavioral difficulties affecting getting to doctor’s appointments. Consistently, the clinician and patient, or patient and other participants of DBT skills training might work on specific abilities, such as *checking the fact* and *problem solving*, to resolve interfering emotions with effective self-care behaviors. Furthermore, other emotion regulation skills, such as *cope ahead* (Linehan, 2014; p. 394), could address behavioral dysregulation that does not allow individuals with BPD engage in behaviors necessary to manage different medical conditions. An additional goal that is considered within the DBT model is to maintain a healthy physical condition long-term in order to reinforce emotional stability over time. Accordingly, the *PLEASE* skill, which refers to an emotion regulation module (Linehan, 2014; pp. 396–397), might be included in reaching this therapeutic goal through the development of a more adaptive and healthy lifestyle. This skill focuses on different domains of self-care, which are identified by the acronym as follows: *PL – treat PhysicaL illness* (i.e., take care of your body. See a doctor when necessary. Take prescribed medication); *E – Balance Eating* (i.e., Try to eat the amounts and kinds of foods that help you feel good. Eat regularly and mindfully throughout the day); *A – avoid mood-Altering substances* (i.e., stay off illicit drugs and alcohol); *S – balance Sleep* (i.e., Try to get the amount of sleep that helps you feel good, usually between 7 and 9 h); *E –* get Exercise (i.e., Do some sort of exercise 5 to 7 days per week for 20 min each time).

Taking together the clinical scenarios mentioned above, the management of physical diseases among BPD individuals treated with DBT might be a relevant target of intervention starting from its early stages, considering their robust functional associations with the core features of BPD. However, severe and chronic medical conditions could also appear during later stages of the therapy when emotional and behavioral dysregulation are resolved. During stage 3 in which the clinician helps the patient to achieve “ordinary” happiness and unhappiness through the resolution of problems, self-care might become the main goal of the therapy. Consistently, the clinician sustains and shapes the problem-solving and acceptance skills of the patient which were acquired during the early stages of intervention (e.g., interpersonal effectiveness skills module — *DEAR MAN, FAST*, and *GIVE* — Linehan, 2014; pp. 248–260), especially in an individual setting. This is in order to solve any difficulty linked to medical conditions (e.g., relational difficulties with doctors, fear of medical treatments or lack of money to access adequate medical assistance).

## Discussion

This perspective paper has sought to highlight the reciprocal relationships between maladaptive emotion regulation strategies and the onset and maintenance of different physical diseases. Starting from a theoretical discussion of this topic, an additional aim of this study was to provide a prototypical clinical scenario to show the functional associations between emotional dysregulation and physical diseases namely in the case of BPD. Considering this clinical population, the current work also sought to show how DBT could be an effective approach to simultaneously address difficulties with emotion regulation and their relationships with medical conditions among individuals with BPD. This theoretical discussion has laid the theoretical background for adapting and empirically implementing the DBT model as an effective psychotherapeutic intervention to prevent the onset of severe and chronic physical conditions in high-risk populations and to manage the maladaptive effects of medical conditions on emotional functioning.

The empirical evidence regarding the role of emotion regulation mechanisms on the onset and maintenance of physical diseases, especially chronic ones (e.g., cardiovascular and metabolic diseases and chronic pain), suggested that the inflexible use of maladaptive emotion regulation strategies linked to attentional functioning (i.e., rumination and worry) together with dysfunctional response-focused emotion regulation processes (i.e., EA and suppression) could have a direct link to medical conditions through a physiological pathway, sustaining altered HPA activity. Maladaptive emotion regulation strategies might also indirectly impact the probability of developing physical diseases through a behavioral pathway, which is characterized by a recurrent engagement in unhealthy behavior (e.g., substance use and binge eating) to suppress or avoid distressing emotional states. However, studies on the effects of several medical diseases on emotional functioning demonstrated that they might be biological vulnerability factors for adaptive emotion regulation taking into account the physiological consequences of physical illness on HPA alterations (McEwen, 2007; Moulton et al., 2015). Moreover, medical conditions might be triggers for the onset and stabilization of depressive and anxious symptoms, which are recognized as epiphenomena of difficulties in regulating negative emotions (e.g., Garnefski and Kraaij, 2006; Cisler et al., 2010) linked to physical diseases.

Therefore, the previous empirical findings suggested that maladaptive emotion regulation mechanisms and medical conditions show complex interrelationships that reciprocally influence and reinforce each other over time. The complexity of the mutual relationships between these conditions finds a prototypical expression among individuals with BPD. Indeed, consistent evidence has confirmed that biological alterations (Crowell et al., 2009; Schulze et al., 2016, 2019) and dysfunctional psychological mechanisms (e.g., emotional suppression and avoidance) (Carpenter and Trull, 2013; Cavicchioli et al., 2015) sustain BPD emotional dysregulation. Difficulties in regulating emotions among individuals with BPD are also manifested in recurring and rigid maladaptive behaviors, which might become comorbid clinical conditions such as SUDs and binge eating disorder. Individuals affected by BPD show significantly higher rates of chronic physical diseases (e.g., cardiovascular and metabolic obesity-related diseases, and chronic pain) than the general population. This is consistent with the role of emotional dysregulation and related behaviors in BPD and with the hypothesis linking these clinical aspects with medical conditions. This high prevalence of chronic illness among individuals with BPD should be considered a relevant biological vulnerability factor of emotional dysregulation (i.e., altered activity of HPA) and as a persistent negative emotional-eliciting situation, which might trigger the disorder’s core maladaptive behaviors (e.g., suicide attempts and non-suicidal self-injury behaviors) (Druss and Pincus, 2000; Singhal et al., 2014). Hence, medical conditions should be carefully considered for a comprehensive examination of BPD emotional and behavioral dysregulation. Accordingly, clinical interventions for BPD should focus on the managing this biological dimension in order to provide appropriate treatments for individuals affected by this disorder.

Taking these considerations together, the DBT model might be a well-validated approach to address these complex clinical issues among individuals with BPD. The principles of FBA, in particular, represent the key assessment tools to precisely evaluate how difficulties with emotion regulation, problematic behaviors, and medical conditions are mutually linked and reinforce each other. By starting from FBA and applying the DBT hierarchical approach, the clinician can identify the primary and secondary targets of intervention on the base of consequences of emotions, behaviors, and medical conditions on the integrity of life, continuity of therapy, and quality of life. After the clinician identifies the clinical priorities, the patient learns a wide set of cognitive behaviors to improve acceptance toward experiences and external events (i.e., mindfulness and distress tolerance skills) and implement effective problem-solving strategies to manage distressing emotions and related situations (i.e., emotion regulation and interpersonal effectiveness skills) during group skills training. The clinician and patient work together in individual psychotherapy to reach a dialectical balance between acceptance and problem-solving skills focusing on specific problematic situations and individualized functional relationships among emotional dysregulation, maladaptive behaviors and the effects of medical conditions on psychological functioning.

The clinical principles of DBT mentioned above have made it possible to effectively adapt this model to treat several conditions among adolescents and adults aside from BPD (e.g., Ritschel et al., 2015; Valentine et al., 2020), which are characterized by high levels of emotional and behavioral dysregulation. Consistently with the flexibility of the DBT model and empirical evidence linking emotional dysregulation to medical conditions, this theoretical and clinical framework might be implemented as a valid prevention program for populations at high-risk (e.g., tobacco smokers and emotional eaters) of developing severe medical conditions (e.g., cardiovascular and metabolic obesity-related diseases). Furthermore, the DBT model could be adapted and carried out as a psychotherapeutic intervention for patients affected by chronic physical illness with a relevant impact on emotional functioning (e.g., chronic pain, diabetes, hypertension, and rheumatoid arthritis) in order to improve the effects of gold-standard pharmacological treatments.

Despite these considerations based on robust empirical evidence, some limitations must be discussed. First of all, future mediational and longitudinal studies on the general population should be carried out in order to empirically support the hypothesis concerning physiological and behavioral pathways linking maladaptive emotion regulation strategies to a heightened risk of developing different medical conditions. This should also be replicated using case-control studies comparing high-risk or clinical populations with low-risk or healthy controls. The mediating role of physiological changes, especially concerning the HPA axis, in maladaptive emotion regulation strategies should be investigated with adequate psychophysiological (e.g., levels of corticototropin-releasing hormone and cortisol) and neuroimaging indexes (e.g., adrenal gland volume) (Golden et al., 2011). Considering comprehensive, valid assessment procedures, future studies should include neuropsychological measures of impulsivity (e.g., GO NO-GO task; IOWA gambling task) (Rochat et al., 2018) in order to support the behavioral pathway linking maladaptive emotion regulation strategies to the onset and maintenance of medical conditions, especially chronic ones. An additional limitation is the absence of empirical data regarding the improvement of co-occurrent physical illness among BPD patients treated with standard DBT or DBT skills training as a stand-alone treatment. Therefore, future clinical trials on the DBT model for the treatment of BPD should include the assessment of medical conditions and their remission as a relevant outcome for testing the efficacy of the intervention. Ultimately, there are no clinical studies regarding the implementation of the DBT model as a structured psychotherapeutic intervention supporting well-validated pharmacological treatments for different medical diseases. Hence, future empirical adaptions of the DBT model are needed to sustain the theoretical considerations discussed in the current work, especially regarding the effectiveness of this clinical framework for long-term remission of several physical illnesses.

## Conclusion

Empirical evidence suggests that physiological alterations and unhealthy behaviors to suppress or avoid emotional states play a role in the mutual relationship between maladaptive emotion regulation strategies and the onset and maintenance of several chronic physical diseases. These functional relationships are particularly expressed among individuals with BPD, who show high levels of emotional and behavioral dysregulation. Accordingly, the DBT model might be considered a well-validated therapeutic approach to address maladaptive associations between emotional dysregulation and medical conditions in this clinical population. However, future clinical trials on DBT should include the stabilization and remission of physical diseases as relevant outcomes for a comprehensive evaluation of this model’s efficacy in treating BPD. Furthermore, future implementation studies of DBT among individuals affected by different physical diseases are needed in order to demonstrate its effectiveness in sustaining the long-term therapeutic effects of well-validated pharmacological treatments for these conditions.

## Author Contributions

All authors alone were responsible for the content and writing of this manuscript. All authors equally participated in manuscript preparation.

## Conflict of Interest

The authors declare that the research was conducted in the absence of any commercial or financial relationships that could be construed as a potential conflict of interest.
